# Knocking-Down *Meloidogyne incognita* Proteases by Plant-Delivered dsRNA Has Negative Pleiotropic Effect on Nematode Vigor 

**DOI:** 10.1371/journal.pone.0085364

**Published:** 2013-12-31

**Authors:** José Dijair Antonino de Souza Júnior, Roberta Ramos Coelho, Isabela Tristan Lourenço, Rodrigo da Rocha Fragoso, Antonio Américo Barbosa Viana, Leonardo Lima Pepino de Macedo, Maria Cristina Mattar da Silva, Regina Maria Gomes Carneiro, Gilbert Engler, Janice de Almeida-Engler, Maria Fatima Grossi-de-Sa

**Affiliations:** 1 Graduate Program in Biology Molecular, Universidade de Brasília, Brasília, Distrito Federal, Brazil; 2 Embrapa Recursos Genéticos e Biotecnologia, Brasília, Distrito Federal, Brazil; 3 Embrapa Cerrados, Planaltina, Ditrito Federal, Brazil; 4 Graduate Program in Genomic Sciences and Biotechnology, Universidade Católica de Brasília, Brasília, Distrito Federal, Brazil; 5 Institut National de la Recherche Agronomique, UMR 1355 ISA/Centre National de la Recherche Scientifique, UMR 7254 ISA/Université de Nice-Sophia Antipolis, UMR ISA, Sophia-Antipolis, France; Ghent University, Belgium

## Abstract

The root-knot nematode *Meloidogyne incognita* causes serious damage and yield losses in numerous important crops worldwide. Analysis of the *M. incognita* genome revealed a vast number of proteases belonging to five different catalytic classes. Several reports indicate that *M. incognita* proteases could play important roles in nematode parasitism, besides their function in ordinary digestion of giant cell contents for feeding. The precise roles of these proteins during parasitism however are still unknown, making them interesting targets for gene silencing to address protein function. In this study we have knocked-down an aspartic (*Mi-asp-1*), a serine (*Mi-ser-1*) and a cysteine protease (*Mi-cpl-1*) by RNAi interference to get an insight into the function of these enzymes during a host/nematode interaction. Tobacco lines expressing dsRNA for *Mi-ser-1* (dsSER), *Mi-cpl-1* (dsCPL) and for the three genes together (dsFusion) were generated. Histological analysis of galls did not show clear differences in giant cell morphology. Interestingly, nematodes that infected plants expressing dsRNA for proteases produced a reduced number of eggs. In addition, nematode progeny matured in dsSER plants had reduced success in egg hatching, while progeny resulting from dsCPL and dsFusion plants were less successful to infect wild-type host plants. Quantitative PCR analysis confirmed a reduction in transcripts for *Mi-cpl-1* and *Mi-ser-1* proteases. Our results indicate that these proteases are possibly involved in different processes throughout nematode development, like nutrition, reproduction and embryogenesis. A better understanding of nematode proteases and their possible role during a plant-nematode interaction might help to develop new tools for phytonematode control.

## Introduction

Sedentary endoparasitic nematodes of the genus *Meloidogyne* (root-knot nematodes, RKN) are pathogenic nematodes causing losses of about 125 billion US$ dollars annually across the world [1]. The species *Meloidogyne incognita* is the most damaging phytonematode in agriculture worldwide [2], mainly due to its polyphagous lifestyle, its wide distribution and high mitotic parthenogenetic rate of reproduction. Root-knot nematodes are obligate parasites that have developed a highly specialized and unique way to infect their hosts. To assist their sedentary life cycle, they inject a plethora of effector proteins into host cells where feeding sites will be formed. These effectors alter the rate of vascular root cell division, resulting in cellular redifferentiation, culminating in the formation of huge sized multinucleate and metabolically active cells known as giant cells [3]. Nematode effectors consist of proteins (i.e. cellulases, proteases, etc) and other molecules of unknown function, (i. e. nematode glands proteins [4,5]) secreted by plant parasites. The mechanical action of the stylet allows the precise and localized deposition of effectors in the host cells. Effectors promote nematode penetration and migration in the plant root and play an important role to overcome plant defenses supporting initiation and maintenance of feeding site development [6].

Proteases are ubiquitous proteolytic enzymes that cleave internal peptide bonds of proteins and peptides. They are present in a diverse range of organisms including bacteria, plants, invertebrates and vertebrates. In the case of helminthic parasites, functions of proteases in host-parasite interactions are very diverse and can range from participation during invasion of host tissues, nutrition of the parasite, and escape from host defense responses [7]. Proteases encountered in the five major classes of nematodes are present in the phytopathogens *M. incognita* and *Meloidogyne hapla* [8]. Proteases predicted from the *M. incognita* genome [9] are the abundantly present metallo proteases, followed by cysteine ​​proteases, serine, aspartic, and threonine proteases. Some proteases previously described in *M. incognita* are: two very similar Cathepsin L (cysteine) proteases [10,11], a chimotrypsin-like serine protease [12] and a cathepsin D aspartic protease [13]. Another aspartic protease was found to be implicated in the process of parasitism of *M. incognita* and showed to be secreted into the plant apoplast [14]. In view of the importance of this ubiquitous class of enzymes involving a wide range of fundamental metabolic functions in host-parasite interactions, these proteases can be considered as important targets for the bio-engineering of novel crop plants with increased tolerance towards nematode parasitism [15].

The discovery of the pathway controlling gene expression through small interfering RNA molecules (siRNA) and microRNAs (miRNA) has opened new avenues to explore gene function and to unravel complex developmental processes [16]. RNA interference (RNAi) is generally accepted as a powerful tool for manipulating gene expression and perform analyses of their functions [17]. RNAi induction upon ingesting double-stranded RNA (dsRNA) during *in vitro* experiments has clearly proven to be sufficiently effective for the nematode *Caenorhabditis elegans*. In contrast, efficient dsRNA intake by plant parasitic nematodes demonstrated to be more difficult. This recalcitrant behavior of phytoparasitic nematodes may be due to the fact that these organisms are obligate parasites depending on a healthy living host in order to feed and develop. In addition, when phytonematodes are outside the plant they do not feed [18], preventing dsRNA uptake.

The first *in vitro* RNAi experiments performed in cyst nematodes made use of the neurotransmitter octopamine to stimulate dsRNA ingestion by J2 pre-parasitic stages of *Heterodera glycines* and *Globodera pallida* [19]. Other studies on root-knot nematode *M. incognita* genes such as proteases, gland proteins and peroxiredoxins, showed efficient gene suppression using the same method [11,20-23]. Also, ingestion of specific dsRNA delivered *in planta*, targeted a gene encoding protein 16D10 expressed in esophageal glands [22] and knocked-down genes of a RNA splicing factor and an integrase [24]. In both cases parasitic nematodes were impaired in their development. Currently, several studies have been conducted expressing dsRNA in plants to characterize gene function [25,26] and to identify genes with potential value to control nematode propagation [27-32]. 

Here we describe the effects of the knock-down of three candidate proteases with potential involvement in parasitism of *M. incognita* using host-delivered RNAi with the aim to gain knowledge on protease function during plant-nematode interaction. We provide expression pattern data of these proteases throughout different nematode stages and illustrate the effects of protease knock-down in galls and developing nematodes as well as the impact on nematode reproduction and progeny virulence.

## Material and Methods

### Nematode culture

*Meloidogyne incognita*, race 3, was propagated and maintained on greenhouse-grown tomato plants (*Solanum lycopersicum* L.). Collection of nematodes was performed at different developmental stages (28 to 90 days after inoculation-DAI of tomato plants). Eggs were extracted according to Hussey and Barker [33] with minor modifications. Tomato roots were grounded in a blender for two minutes in sodium hypochlorite (NaOCl) 0.5%. Egg counting was done microscopically using a Peters slide [34]. To collect pre-parasitic second-stage juveniles (ppJ2), egg suspension was subjected to modified Baermann funnel technique and kept at room temperature in a recipient containing distilled water to enable egg hatching and subsequent nematode collection. Collection of hatched J2’s was performed every other day during a week. To collect parasitic juveniles and females, infected roots were incubated in a solution of 25% (v / v) pectinase for 16 hours. Then roots were rinsed in tap water in a 100 mesh sieve, and were pelleted by centrifugation at 2500 *g* for 10 min in a suspension of kaolin (inert substrate). Parasitic juveniles and females were resuspended in 40% (w / v) sucrose by centrifugation and precipitated as mentioned above. Thereafter nematodes were deposited on a 100 mesh sieve, washed in distilled water and transferred into a container, where manual collection was performed.

### Expression pattern of *Meloidogyne incognita* proteases

The search for expressed sequence tags (ESTs) from *M. incognita* proteases genes in NCBI-dbEST (http://www.ncbi.nlm.nih.gov/dbEST) was performed using keywords search and BLASTx [35], available at NCBI ( http://blast.ncbi.nlm.nih.gov/Blast.cgi) using all the ESTs available for *M. incognita*. 

To quantify the expression of proteases *Mi-asp-1*(Accession: DQ360827) , *Mi-ser-1* (AY714229) and *Mi-cpl-1*(AJ557572) total RNA from *M. incognita* eggs, pre-parasitic J2 and mature females was extracted using the RNeasy Mini Kit (Qiagen, USA) following the manufacturer's instructions. The extracted RNAs were eluted in water and stored at - 80 °C until use. The complementary DNA (cDNA) was produced using First-Strand cDNA Synthesis kit (Invitrogen, USA) from total RNA egg, pre-parasitic J2, parasitic juvenile or female *M. incognita*, thereafter, all cDNAs were stored at -20 °C. Amplification and detection were performed in the 7500 Fast Real-Time PCR System (Applied Biosystems). Reaction mixtures contained a final volume of 10 µL, containing 5 µL of SYBR Green PCR Mix (LGC), 2 µL of cDNA, 2.6 µL of double distilled H_2_O and 0.2 µM of each primer (Table 1) and 2 µL of 50-fold diluted cDNA templates. PCR conditions were as follows: 95°C for 10 min, followed by 40 cycles of 95°C for 15 s and 60°C for 1 min. At the end of the program a melting curve for each primer (60-94°C read every 0.5°C) was acquired to ensure that only single products were generated. The *M. incognita* 18S ribosomal subunit (mi18S) was used for normalization of qRT-PCR data (Table 1). This gene was previously identified as showing constant expression in similar experiments in *M. incognita* [36]. Raw data were treated using the online software qPCR miner (http://www.miner.ewindup.info) [37], to find and assess Ct values and primers efficiency. The relative expression of each gene was calculated according to the method of Pfaffl [38] using the program QBASE plus (Biogazelle, Belgium). The primer list is given in Table 2. Two independent quantitative RT-PCR reactions were carried out per sample and two biological replicates were performed.

**Table 1:** List of primers used for transcription evaluation during different stages of *Meloidogyne incognita* development by qRT-PCR.

**Primer name**	**Target gene (accession number)**	**Sequence (5'-3')**	**Target region (bp)**	**Amplicon size (bp)**	**Primer Reference**
MiASPqPCR F	*mi-asp-1* (DQ360827)	AATTGGAGGTCATTCATACG	1026	107	This study
MiASPqPCR R	*mi-asp-1*	GTGGAAGATCAATTCCCATA	1132	-	This study
MiSERqPCR F	*mi-ser-1* (AY714229)	CATTTTCCGACCTTGCACTT	559	157	This study
MiSERqPCR R	*mi-ser-1*	GGTCGGTCATTGAGCAAACT	715	-	This study
MiCISqPCR F	*mi-cpl-1* (AJ557572)	TGTACACTTTGCTTGTCGAG	31	103	This study
MiCISqPCR R	*mi-cpl-1*	GAATTTCTTCGAGATCGTTG	133	-	This study
Mi18S F	*mi-18S* (U81578)	ACCGTGGCCAGACAAACTAC	852	114	[36]
MI18S R	*mi-18S*	GATCGCTAGTTGGCATCGTT	966	-	[36]

**Table 2:** List of primers used for gene for *M. incognita* protease gene fragment cloning and transgenic tobacco genotyping.

**Primer name**	**target gene**	**Sequence (5'-3')**	**Target region (bp)**	**Amplicon size (bp)**
Mi-ASP1_GTWY_Fw	*mi-asp-1*	*(ATTB1)-**TCTTTATAGGCCGTTACTACACTG**	1165	198
Mi-ASP1_GTWY_Rv	*mi-asp-1*	**(ATTB2)-**AAGCAATTTCAATAAAATCATCAG**	1363	-
Mi-SER_GTWY_Fw	*mi-ser-1*	*(ATTB1)**-AATTTCCTGTTATCTGCTGCCCAC**	326	210
Mi-SER_GTWY_Rv	*mi-ser-1*	**(ATTB2)**-TTTTGTTTTATAATAACGAGAGAG**	536	-
Mi-CIS_GTWY_Fw	*mi-cpl-1*	*(ATTB1)**-CCAATTTCTGTAGCAATTGATGCC**	888	201
Mi-CIS_GTWY_Rv	*mi-cpl-1*	**(ATTB2)**-TCGAATATACCCATTTTCTCCCCA**	1089	-

^*^ (ATTB1): GGGGACAGTTTGTACAAAAAAGCAGGCTTG

^**^ (ATTB2): GGGGACCACTTTGTACAAGAAAGCTGGGTG

### Vector cloning for RNAi knockdown experiments

Selected fragments from genes of three proteases (ASP-1, SER-1 and CPL-1) were cloned from *M. incognita* cDNA into pGEM-T easy vector (Promega, USA). Clones were checked through DNA sequencing. We purchased a plasmid containing the three fragments fused in tandem (ASP-SER-CPL), named FUSION, to test the effect of simultaneous knocking down of proteases. This fragment was synthesized and subcloned into the vector pBlueScript II (Agilent Technologies, USA) by Epoch Biolabs (Sugar Land, TX, USA).

To obtain constructs for expression of dsRNA specific to *M. incognita* proteases sequences, primers were designed (Table 2) containing attB1and attB2 sites required for cloning into vectors using the Gateway^®^ method (Invitrogen, USA). All fragments and fusion were subcloned into the pDONR vector™ 221, using BP clonase (PCR Cloning System with Gateway^®^ Technology Kit, Invitrogen, USA), and then transferred again by recombination, using LR clonase enzyme, for the binary vector pK7GWIWG2 (I) [39] used for expressing dsRNA in plants.

### Generation and selection of transgenic RNAi tobacco lines

dsRNA constructs were inserted into *A. tumefaciens* strain GV3101 using a standard electroporation method and plated on LB medium containing rifampicin (100 µg ml^-1^), kanamycin (50 µg ml^-1^) and streptomycin (300 µg ml^-1^). Transformation of tobacco leaves (*N. tabacum* var. SR1) was carried out according to the protocol described by Gallois and Marinho [40]. 

Seeds of the T0 generation were collected and stored until use. For selection of transformed plants, seeds were surface sterilized by incubation in 70 % ethanol for 5 min, 1% sodium hypochlorite for 2 h, and four times washed with sterile water. Then, they were plated on MS medium [41] supplemented with kanamycin (100 mg/l).

Germinating and developing seeds under kanamycin selection were genotyped in a pool of five seedlings using the Extract-N-Amp Plant PCR kit (Sigma-Aldrich, USA). We amplified three different DNA fragments from transformed plants: one fragment corresponding to the one selected for transformation without discriminating the position in the vector, and two corresponding to sense and anti-sense fragments (related to the position in the vector) (Table 2 and 3). Then, total RNA was extracted from a pool of whole seedlings, five and ten days after germination, for each transformation event using the RNeasy Plant Mini Kit (Qiagen, Valencia, CA, USA). RNA obtained was used to analyze the trangene expression of intron of hairpin dsRNA structure formed by pK7GWIWG2(I) vector of each construct, using the methodology adapted from Patel et al [26]. This analysis was performed by RT-PCR using First-Strand cDNA Synthesis kit (Invitrogen, USA) as described by the manufacturer, using 1 µg of total plant RNA. The oligonucleotide primer specific for the intron of vector pK7GWIWG2 (I), PK7-Intron-RT-R (Table 3) was used to synthesize the first cDNA strand for each transformation event, and these cDNAs were used as template for amplification of a 150 bp fragment using the oligonucleotide PK7-Intron- Fand Intron_PK7_RV (Table 3).

**Table 3:** List of primers used for transgenic tobacco genotyping and RT-PCR for confirmation of dsRNA cassete transcription.

**Primer name**	**target gene**	**Sequence (5'-3')**	Target region (bp)*	**Amplicon size (bp)**
pK7-Intron – F	Intron pK7GWIWG2(I)	GAGTATAAACTCATTAACTAA	104	145**
Intron_pK7_RV	Intron pK7GWIWG2(I)	TGGCATAGGGGTTTAGATGC	248	--
Intron_pK7_FW	Intron pK7GWIWG2(I)	TAACTCAGCACACCAGAGCA	515	--
pK7-Intron-RT-R	Intron pK7GWIWG2(I)	ATGGAAATGATGAGGTAAGGTTTC	630	--

^*^ Considering only the intron region of the vector pK7GWIWG2(I)

^**^ Amplicon generated by primers pK7-Intron – F and Intron_pK7_RV

After confirming the presence of the transgene by PCR and the fragment of the intron vector by RT-PCR, remaining seedlings were transferred to plastic pots containing soil and acclimatized in greenhouse. Acclimatized plants were genotyped individually by PCR using the Extract-N-Amp Plant PCR kit. Plants, which were genotyped and confirmed to express hairpin dsRNA were used in further bioassays with *M. incognita*.

### Morphological analyses of *M. incognita* induced-galls on tobacco roots expressing hairpin dsRNA

Tobacco T1 seeds from all constructs (Control, dsFusion, dsCPL and dsSER plants) were grown and selected in MS media supplemented with kanamycin 100 µg/ml. After germination, plantlets were transferred to 300 ml pots containing soil with a 16h/8h light/darkness photoperiod at 22°C/20°C (light/dark), respectively. Plant roots were inoculated with 200 J2 per plant. Galls were collected at 14DAI and fixed during one week in 2% glutaraldehyde in 50 mM PIPES buffer, pH 6.9, and subsequently dehydrated and embedded in Technovit 7100 (Heraeus Kulzer) as described by the manufacturer. Embedded gall tissues were sectioned (5 μm) and stained in 0.05% toluidine blue and mounted in Depex (Sigma-Aldrich). Microscopic observations were performed using bright-field optics and images were obtained with a digital camera (AxioCamHRc, Zeiss).

### Nematode staining in galls and image analysis

Control and transgenic tobacco plants (dsFusion, dsCPL and dsSER plants) were treated as described below. Infected roots were collected from the pots 28 DAI and soaked in 1% bleach for 2 min for clearing and permeabilization. After rinsing in water, roots were boiled in acid fuchsin (350 mg stain in 1 liter of 25% acetic acid) for 3 min, rinsed in water, and transferred to the acidified glycerol for examination and dissection according to Atkinson et al. [42]. Nematodes were dissected from galls using 0.6-mm needles and mounted in a Petri dish in a drop of acidified glycerol. Length, area, and roundness measurements were carried out after acquiring images (Axiocam, Zeiss) from 45 randomly-picked nematodes of all treatments, using the AxioVision Image analysis tool (Zeiss). This experiment was repeated twice.

### Infection and reproduction analyses of *M. incognita* on tobacco expressing hairpin dsRNA

Fifteen days after placing selected plants in soil, 18 to 20 plants from each transformation event were used for bioassays. Half of the plants were inoculated with 400 J2 per plant (to count number of galls and egg masses) and half with 2000 J2 per plant (to count number of eggs per gram of root). Plants were kept in a greenhouse under appropriate growth conditions. Six weeks after inoculation, roots from each plant were removed from soil and processed. Extraction of eggs was done according to Hussey and Barker [33]. After egg harvesting, they were allowed to hatch during 15 days, performing counts every 3 days. This bioassay was repeated twice. This scheme allowed us to evaluate number of galls, egg masses, eggs per gram of root and the egg hatching rate.

Furthermore, we infected untransformed tobacco plants with J2s originating from the transgenic plants, as described by Dubreuil et al. [36]. The number of inoculated J2 was 800 per plant. Five to six plants were used for each treatment. This assay was repeated twice and the number of galls and egg masses per plant was recorded. For statistical analysis, all data obtained was normalized with the control treatment to allow data comparison between biological replicates. This standardization was necessary to avoid misinterpretation of results on nematode infection [20,28].

 All data obtained were statistically analyzed by SPSS (SPSS Inc., Chicago, IL, USA) using one-way ANOVA, and Tukey’s test to compare the means. Groups marked by a different letter indicate a significant difference at *P* < 0.05.

### Quantitative RT-PCR (qRT-PCR) of protease genes in *M. incognita* infecting dsRNA-expressing tobacco lines

Tobacco plants confirmed to express hairpin dsRNA were germinated on MS media, were transplanted to 300 ml plastic cups containing soil and placed in a greenhouse. Five to seven plants for each event and 2000 J2 pre-parasitic were inoculated per plant. Six weeks after root inoculation the eggs were processed and extracted according to Hussey and Barker [33].

For each event, *M. incognita* eggs were pooled and grounded in liquid nitrogen with mortar and pestle, and total RNA was extracted using the RNeasy Mini Kit (Qiagen, USA). RNA was treated with Turbo DNase I (Ambion, USA) according to the manufacturer's instructions. First cDNA strand synthesis was done using SuperScript ® III First-Strand Synthesis Supermix for qRT-PCR (Invitrogen, USA) using 500 ng of total RNA. To perform qRT-PCR and analysis we used the same procedure described before.

## Results

### In silico analysis of transcript representation of *Meloidogyne incognita* proteases in EST databanks

**Figure 1:**
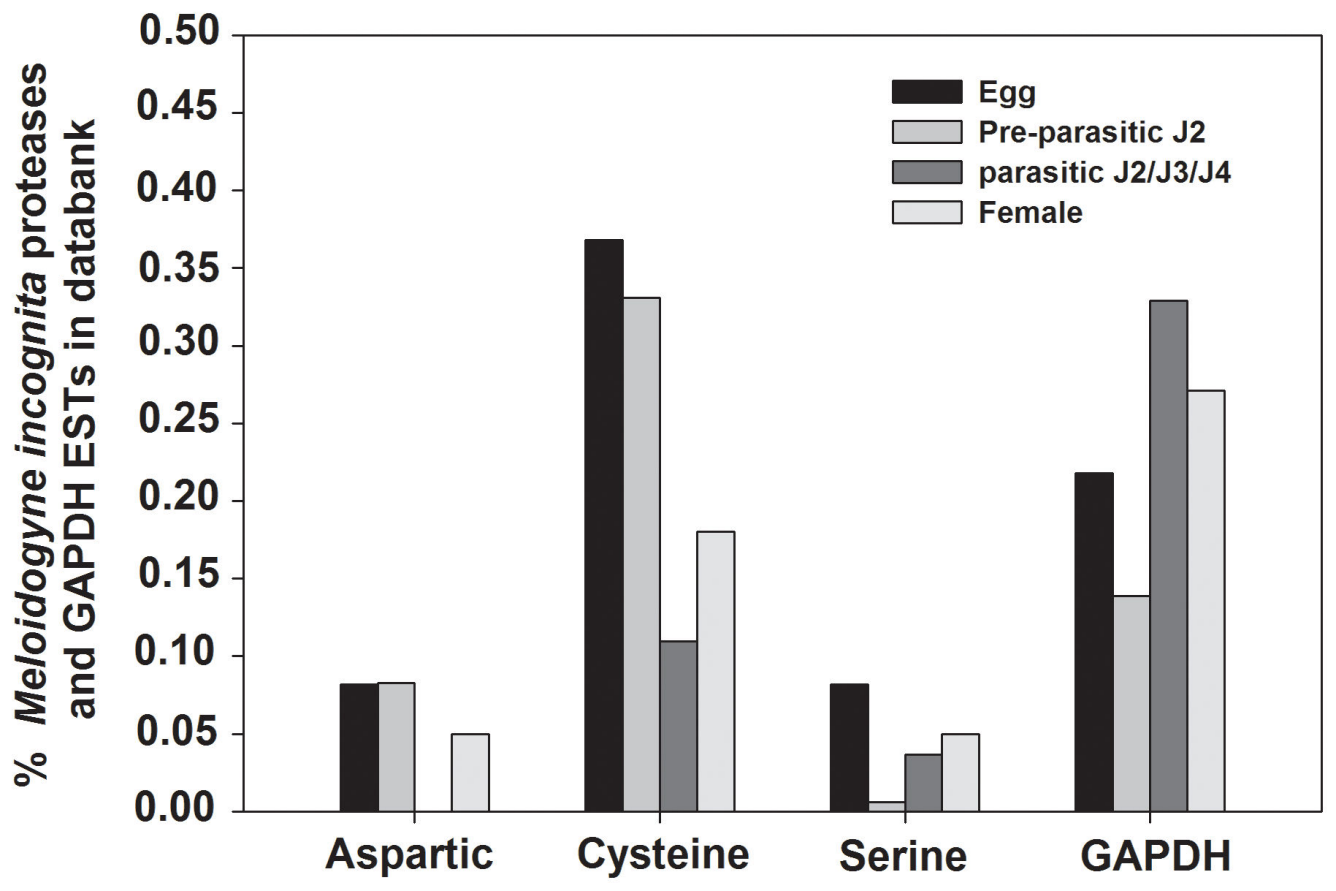
*In* *silico* analyses of all *Meloidogyne incognita* aspartic, serine and cysteine proteases ESTs present in EST data bank dbEST. Representation of *M. incognita* proteases expressed sequence tags (ESTs) in databanks. Bars show the percentage of proteases EST number relative to the total number of EST available for each developmental stage. ESTs from proteases were retrieved from NCBI-dbEST (http://www.ncbi.nlm.nih.gov/dbEST/index.html) and their representation was assessed by the number of ESTs relative to the total number of ESTs available for the developmental stage considered. The developmental stages considered were; eggs (14,671 ESTs), freshly hatched J2s (33,835 ESTs), mixed parasitic stages (3,133 ESTs) and females (4,427 ESTs). The distribution of glyceraldehyde 3-phosphate dehydrogenase (GAPDH) ESTs is indicated for comparison.

Initially, an EST databank survey was done to identify the expression profile of all *M. incognita* aspartic, serine and cysteine proteases. We have searched for proteases ESTs from *M. incognita* named dbEST (http://www.ncbi.nlm.nih.gov/dbEST/index.html) at the NCBI databank. In this bank there are 63,838 ESTs (March, 2013) for *M. incognita*, being 14,671 from eggs, 33,835 from fresh hatched J2, 7,772 from stressed J2, 788 from mixed parasitc J2/J3, 399 from J3-enriched only, 1946 from mixed J3/J4 and 4,427 from adult female. We used the distribution of glyceraldehyde 3-phosphate dehydrogenase (GAPDH) ESTs for comparison, and not data from J3-enriched ESTs since it lacks GAPDH ESTs. A search for proteases ESTs was made against non-redundant protein sequences (NRdb-NCBI) for Nematoda (taxid: 6321), using BlastX with a cutoff of E-value < 10^-10^. *In silico* analysis of proteases transcript represented in EST databanks suggested a higher expression level of cysteine protease genes in all nematode stages when compared with aspartic and serine proteases (Figure 1). Detailed information about the number of ESTs per protease class is described in Table S1.

### Selection and transcription pattern of *Meloidogyne incognita* proteases

To better understand protease functioning in the *M. incognita* phytoparasitism, one gene for each of the three catalytic classes of proteases have been selected for detailed analysis. The selected genes were an aspartic protease cathepsin D type, *Mi-asp-1* (Accession: DQ360827), a chymotrypsin-like serine protease, *Mi-ser-1* (AY714229), and a cysteine ​​protease cathepsin L type, *Mi-cpl-1* (AJ557572). Aspartic and serine proteases have been previously isolated by RT-PCR, 5 'and 3' RACE from a cDNA library of *M. incognita* in our laboratory [12,13]. The cysteine ​​protease studied was isolated from cDNA J2 according to Neveu et al. [10]. We have confirmed the presence of ESTs for these genes in almost all stages of nematode development (Table 4) and the cysteine protease *Mi-cpl-1* has the highest number of ESTs.

**Table 4:** Number of ESTs in databank (dbEST) for protease genes analysed.

	**Protease genes**		
**Stage of development**	*Mi-asp-1*	*Mi-cpl-1*	*Mi-ser-1*
Egg	10	30	7
pre-parisitc J2	10	16	0
parasitic J2/J3/J4	2	1	0
Female	2	6	1
Total	24	53	8

To determine whether *M. incognita* regulates the expression of these proteases, the amount of transcripts for each stage of nematode development was quantified by qRT-PCR. Total RNA was extracted from eggs, pre-parasitic J2 (ppJ2), parasitic juveniles (pJ2/J3/J4) and adult female. 

**Figure 2:**
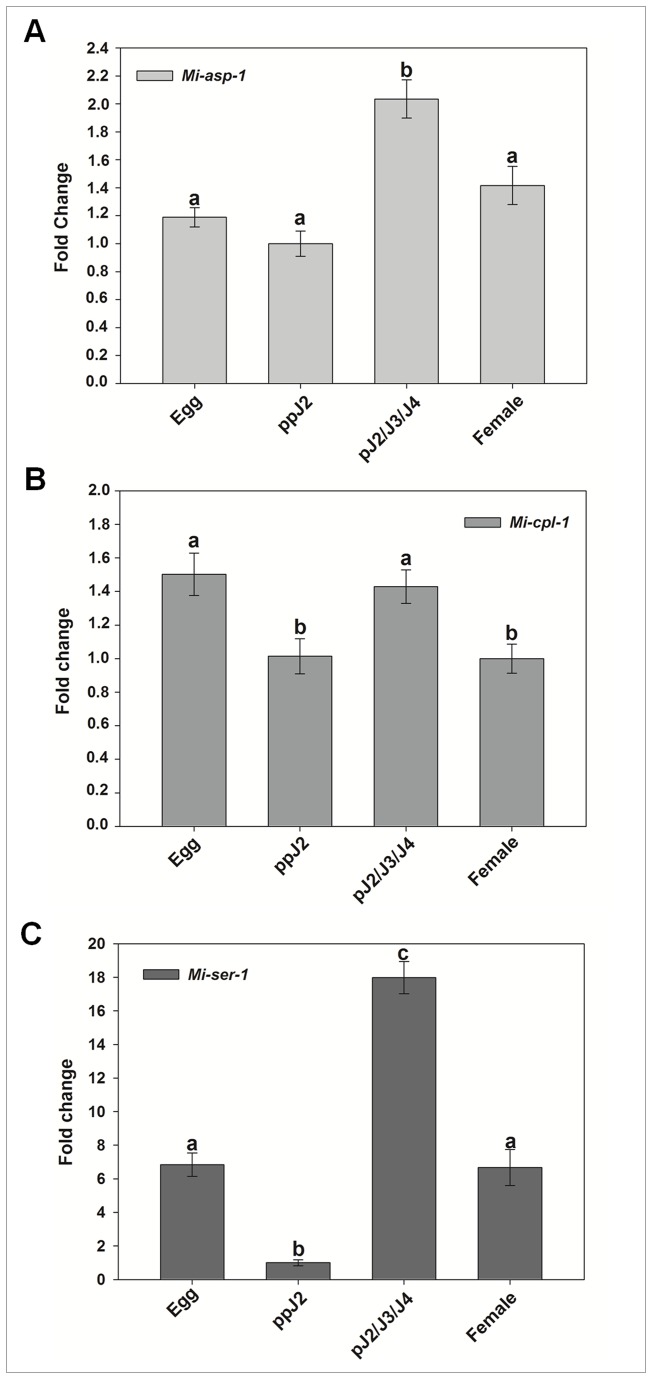
Relative abundance of specific protease gene transcripts in *Meloidogyne incognita*. Real-time qRT-PCR analysis of *M. incognita* proteases transcript levels at different stages of the nematode life cycle. (A) Cathepsin D-like aspartic proteinase (*Mi-asp-1*, Accession: DQ360827). (B) Chymotrypsin-like serine proteinase (*Mi-ser-1*, AY714229). (C) Cathepsin L cystein proteinase (*Mi-cpl-1*, AJ557572). Each bar represents the mean of duplicate assays repeated twice. Standard errors are shown. Different letters mean statistical difference (*p*≤0.05) according to the iteration test (Rest 2009 Software). The results are presented as fold change in comparison to the stage that had the smaller relative expression value that was arbitrarily designed as 1.

Transcript accumulation of *Mi-asp-1* was twice more in parasitic juveniles (pJ2/J3/J4) compared to pre-parasitic J2 (ppJ2) (Figure 2A), while expression levels between egg, ppJ2 and female were statistically the same. Increased accumulation of transcripts observed during stages that nematodes are actively feeding on the host plant indicates the possible involvement of the aspartic protease in the process of parasitism. 

When transcript levels were assessed of *Mi-cpl-1*, values were very close for all nematode stages, but with significant differences amongst them (Figure 2B). Transcript levels from egg and parasitic J2/J3/J4 are statistically similar but different from pre-parasitic J2 and female. Higher transcript levels in egg and parasitic J2 than in pre-parastic J2 and females suggest a possible implication in processes of egg maturation, moulting from J1 to J2 or participation in infection processes when the nematode is within the root.

 When the level of serine protease gene (*Mi-ser-1*) transcripts was evaluated, considerable abundance differences among the four studied nematode stages were observed (Figure 2C). Eighteen times higher transcript level was found in parasitic juveniles (pJ2/3/4) than in pre-parasitic J2 while 7 times more transcripts was found in eggs and females than in pre-parasitic J2. This enzyme could therefore play different roles in nematode biology, like embryogenesis and/or participate in the nematode feeding process.

### Generation of tobacco transgenic lines expressing dsRNA for *M. incognita* proteases

**Figure 3:**
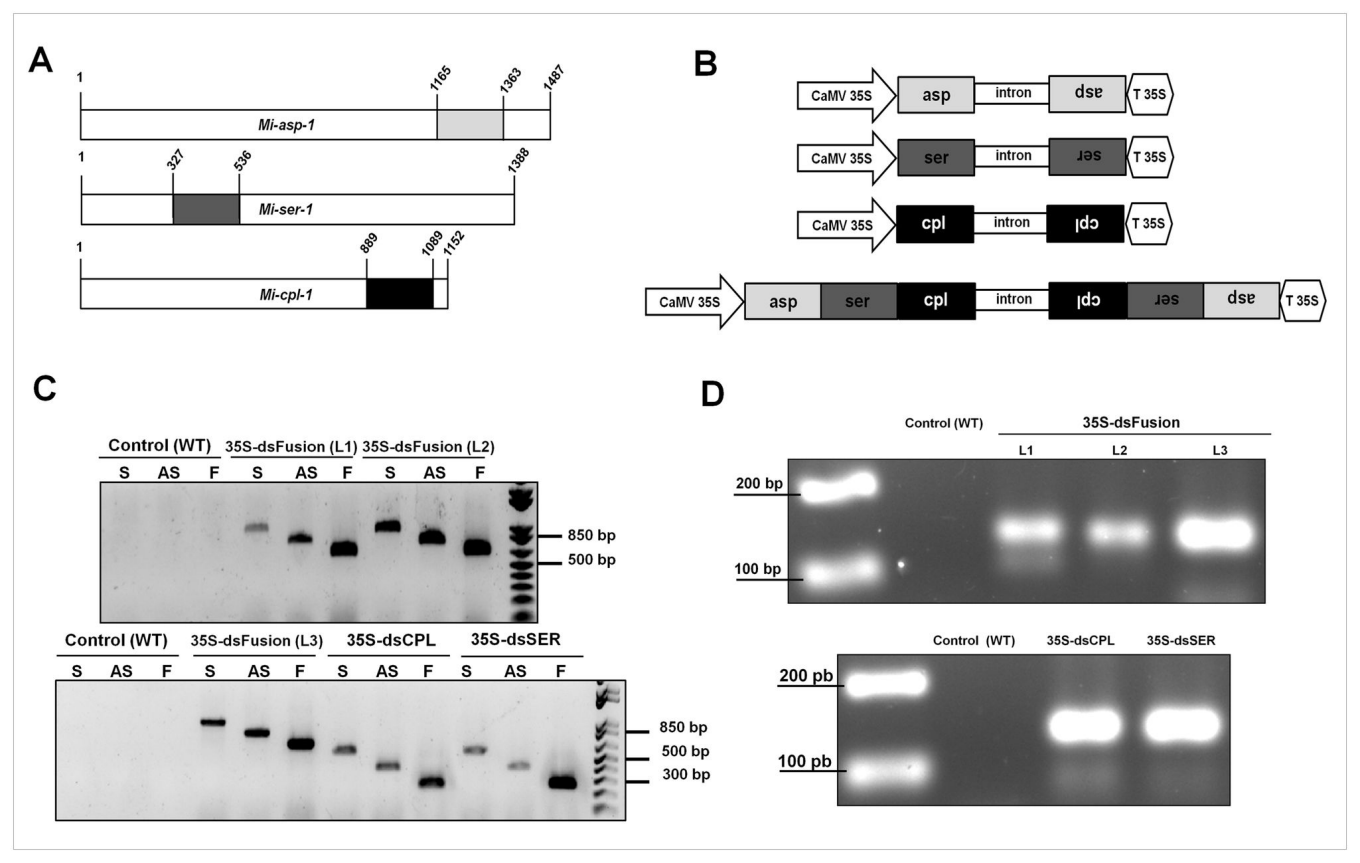
Gene cloning and transgenic tobacco plant generation for host-derived RNA-interference of *Meloidogyne incognita* proteases. (A) Regions of proteinases genes used in RNAi experiments. Numbers indicate nucleotide positions. (B) Schematic representation of the pK7GWIWG2(I) (Karimi et al. 2002) hairpin double-stranded RNA (dsRNA) constructs containing the sense and antisense coding regions fragments of *Mi-asp-1*, *Mi-ser-1*, *Mi-cpl-1* separately and together. (C) Characterization of RNAi lines for silencing of *Mi-ser-1*, *Mi-cpl-1* and the fragments in tandem of *Mi-asp-1*, *Mi-ser-1* and *Mi-cpl-1* (Fusion), by PCR. Attempts for generate ds-*Mi-asp-1* lines were not successful. Sense (S) fragment, anti-sense (AS) fragment, undistinguishable fragment (Sense or Anti-sense) (F). (D) RT-PCR of the single-stranded pK7GWIWG2(I) intron (spacer) of the hairpin dsRNA was used to confirm the expression of *Mi-ser-1*, *Mi-cpl-1* and fusion dsRNAs in seedlings of independent transgenic tobacco lines at 15 d post-germination.

To select regions of *Mi-asp-1*, *Mi-ser-1* and *Mi-cpl-1* of *M. incognita*, these gene sequences were compared with the database (GenBank) using the tool BlastN against non-redundant (nr) databank [35]. To check if there was any chance of off-target effect for homologous genes in the genome of *M. incognita*, a search was made within the genome using again BlastN (http://www7.inra.fr/meloidogyne_incognita/genomic_resources). Specific regions were selected with an E-value = 1e^-05^ as cut-off, for both searching at GenBank database against the nr, and searches within the *M incognita* genome. Chosen regions of *Mi-asp-1* (199 pb), *Mi-ser-1* (210 bp) and *Mi-cpl-1* (201 bp) are shaded in Figure 3A. These regions have no similarity with any sequence at nr database or with any other homologous gene of *M. incognita* genome. 

After tobacco transformation with the three constructs (Figure 3B) via *A. tumefaciens* and tissue culturing, T0 generation plants were acclimated in a greenhouse to obtain T1 seeds. Seeds were germinated in the presence of kanamycin and T1 generation survival rate of 3:1 for all events, corresponding to a Mendelian segregation. Attempts to introduce the 35S-dsASP construct in tobacco were not successful after four transformation trials.

Initial genotyping was carried out with a pool of seedlings, and three events were obtained for 35S-dsFusion, one for 35S-dsCPL, and one for 35S-dsSER. We detected, by PCR, the presence of fragments, sense and antisense genes in all events (Figure 3C), confirming the complete integration of the full dsRNA constructs into the genome of *N. tabacum*. As expected, in control (WT- SR1) plants no fragment was observed. 

The expression of the dsRNA constructs was evaluated in all genomic PCR-confirmed events by RT-PCR of pK7GWIWG2 (I) vector intron. This intron forms a single-stranded loop of the hairpin dsRNA structure and is spliced out during hairpin dsRNA processing, like the PDK intron from pHANNIBAL vector [40]. In all tested lines the 150 bp fragment was detected (Figure 3D). As expected, in control (WT) plants no amplicon was detected.

Hereafter, all experiments were conducted using T1 lines for all constructs. These lines were properly selected by germination in the presence of antibiotic and genotyped by PCR. Three lines generated for the 35s-dsFusion were mixed equally and always used as a pool for all experiments involving nematode infection.

### Expression of dsRNA for proteases did not affect gall development

The effect of over expression of dsRNAs for three protease genes during nematode feeding site development (NFS) was monitored and results compared with galls of control plants. Galls were analyzed 14 DAI when giant cells are expanding, to better evaluate the occurrence of possible differences. No obvious differences were observed in gall morphology, including giant cells and neighboring cells (Figure S1A). Size of giant cells was measured on at least 22 galls per transgenic line, but no size differences were observed (Figure S1B) applying one-way ANOVA (F_3,100_=1.033; *p*=0.381). These results suggest absence of a significant role for these proteases for NFS formation.

### Plants expressing dsRNA against proteases affected the ratio of young and mature females

**Figure 4:**
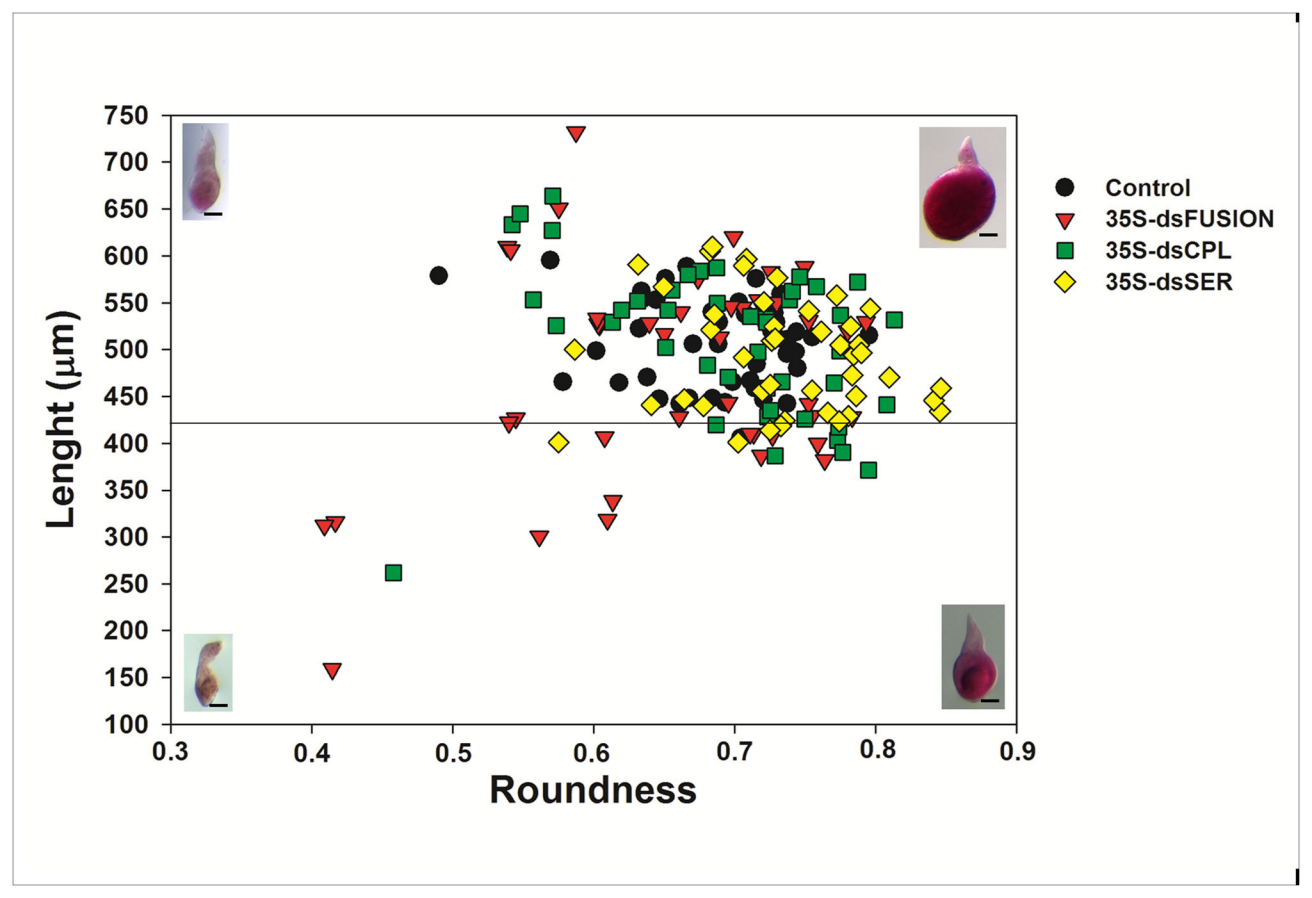
*Meloidogyne incognita* morphology (biometry) 28 days after inoculation (DAI) that matured on transgenic plants expressing dsRNA nematode proteases. Plot showing the size and morphology of *Meloidogyne incognita* at 28 DAI. The length and roundness are indicated by the vertical and horizontal axis, respectively. This plot allows assignment of individual nematodes within two classes: young female (lower quadrant) and mature female (upper quadrant) according to Atkinson et al. [40]. Representative fuchsin stained nematodes are also shown (bar = 100 µM) illustrating examples in shape and size of the two classes.

Figure 4 shows data comparing the size and shape of nematodes at 28 DAI that were fed on plants expressing dsRNA for *Mi-cpl-1*, *Mi-ser-1* and for the Fusion construct (fragments fusion of *Mi-asp-1*, *Mi-cpl-1* and *Mi-ser-1*). Normally at 28 DAI, most nematodes will reach a stage of mature female, laying eggs, with few young females. 

Images of females were analyzed according to Atkinson et al. [42], with some modifications. We measure roundness (R) or circularity, area (A), length (L) and the perimeter (p) of 45 randomly dissected females from each treatment at 28DAI. We calculated the roundness using the formula R = 4πA/p^2^, which is the inverse of the formula previously used [42] to restrict values between 0 and 1. 

All nematodes evaluated were considered female, with R>0.327 (1/3.06, in the calculation by [42]). We use the length L = 422 µm as a limit to differentiate young (L< 422 µm) from mature females (L > 422 µm) [42] (Figure 4). The number of young and mature females was significantly different for nematodes excised from dsFusion plants (Table 5) compared with control treatment (*p*< 0.001) applying chi-square analysis. However, despite a slightly higher proportion of young females excised from dsCPL and dsSER, the difference was statistically not different from controls.

**Table 5:** Percentage of measured nematode females of each dsRNA expressing plant line.

	**n**	**Young female (%)**	**Mature female (%)**
**Control**	45	4.45	95.55
**35S-dsFusion**	45	33.33	66.67 *
**35S-dsCPL**	45	15.55	84.45
**35S-dsSER**	45	8.89	91.11

For all treatments, 45 individuals were analyzed. ^*^ Significant difference in proportions (Chi-square test, *p*<0.001)

### Expression of protease-dsRNAs decreases the number and viability of eggs

**Figure 5:**
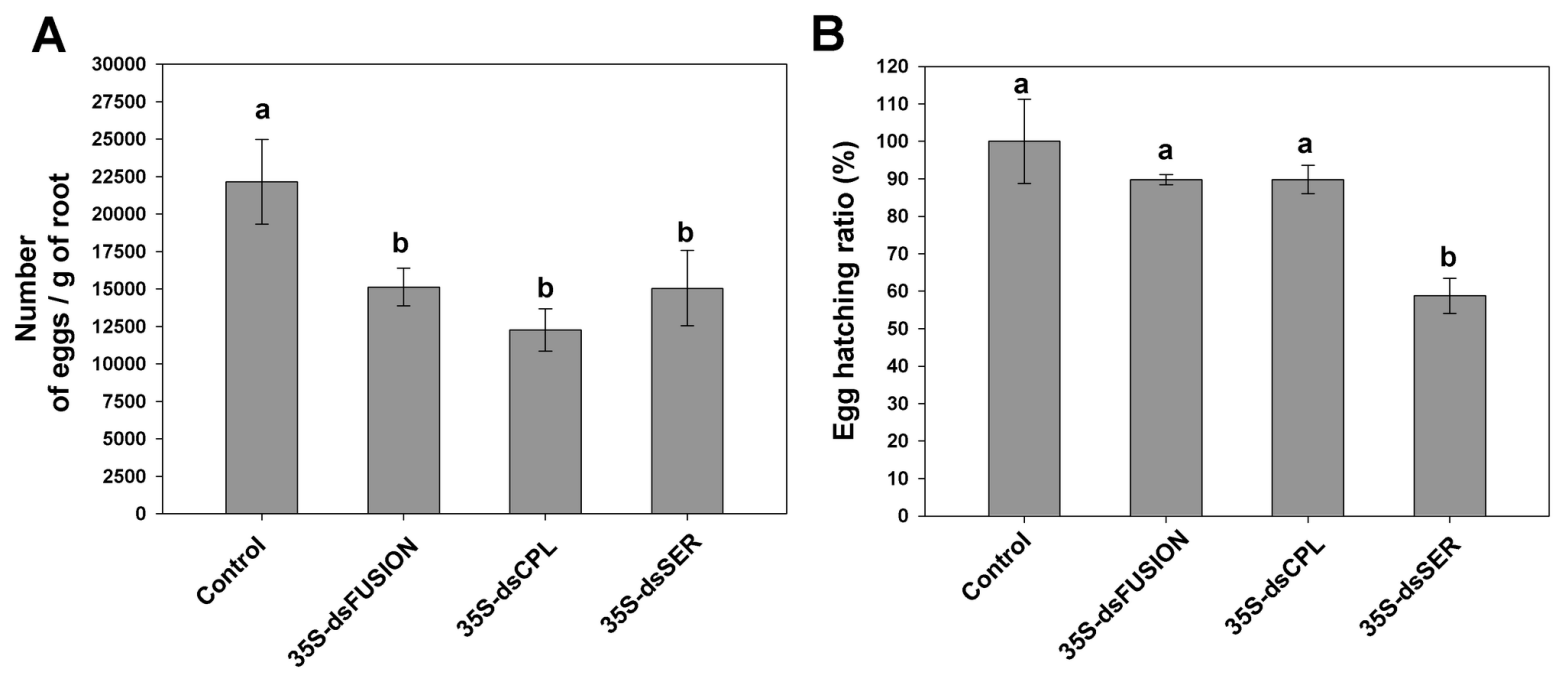
Knock-down of *Meloidogyne incognita* proteases affects nematode reproduction and egg viability (after 45 DAI). (A) Number of eggs per gram of root. (B) Total egg hatching ratio. Experiments were repeated twice. Different letters mean statistical significance through one-way ANOVA and Tukey test (*p*≤0.05).

To investigate the effect of knocking-down protease genes on nematode development and reproduction, bioassays were performed with inoculation of pre-parasitic J2 in plants expressing dsRNA complementary to the nematode proteases. No significant difference in number of galls (Figure S2A) (ANOVA, F_3,56_=2.341, *p*=0.083) or egg masses (Figure S2B) (ANOVA, F_3,56_=1.126, *p*=0.057) was observed for the three transgenic lines (35S-dsFusion, 35S-dsCPL and 35S-dsSER). These data indicate that nematode feeding on plants expressing proteases dsRNAs did not affect gall formation nor impairs females for egg-laying. However, the number of eggs per gram of root was less in plants expressing dsRNA than in the control plants. This reduction reached 33% for dsFusion plants, 42% for dsCPL plants and, 30% for dsSER plants (Figure 5A) (ANOVA, F_3,57_=7.303, *p*=0.001), showing that number of eggs per egg mass significantly decreased. Egg hatching ratio, (number of hatched-J2/number of eggs) analysis, was similar for the dsFusion and dsCPL plants compared to control plants (Figure 5B) (ANOVA, F_3,12_=7.79, *p*=0.04). However, egg hatching ratio in dsSER plants decreased almost 40% suggesting the involvement of *Mi-ser-1* protease on nematode embryogenesis.

### Protease transcript levels decreased in eggs laid by dsRNA-plants-fed-nematode

**Figure 6:**
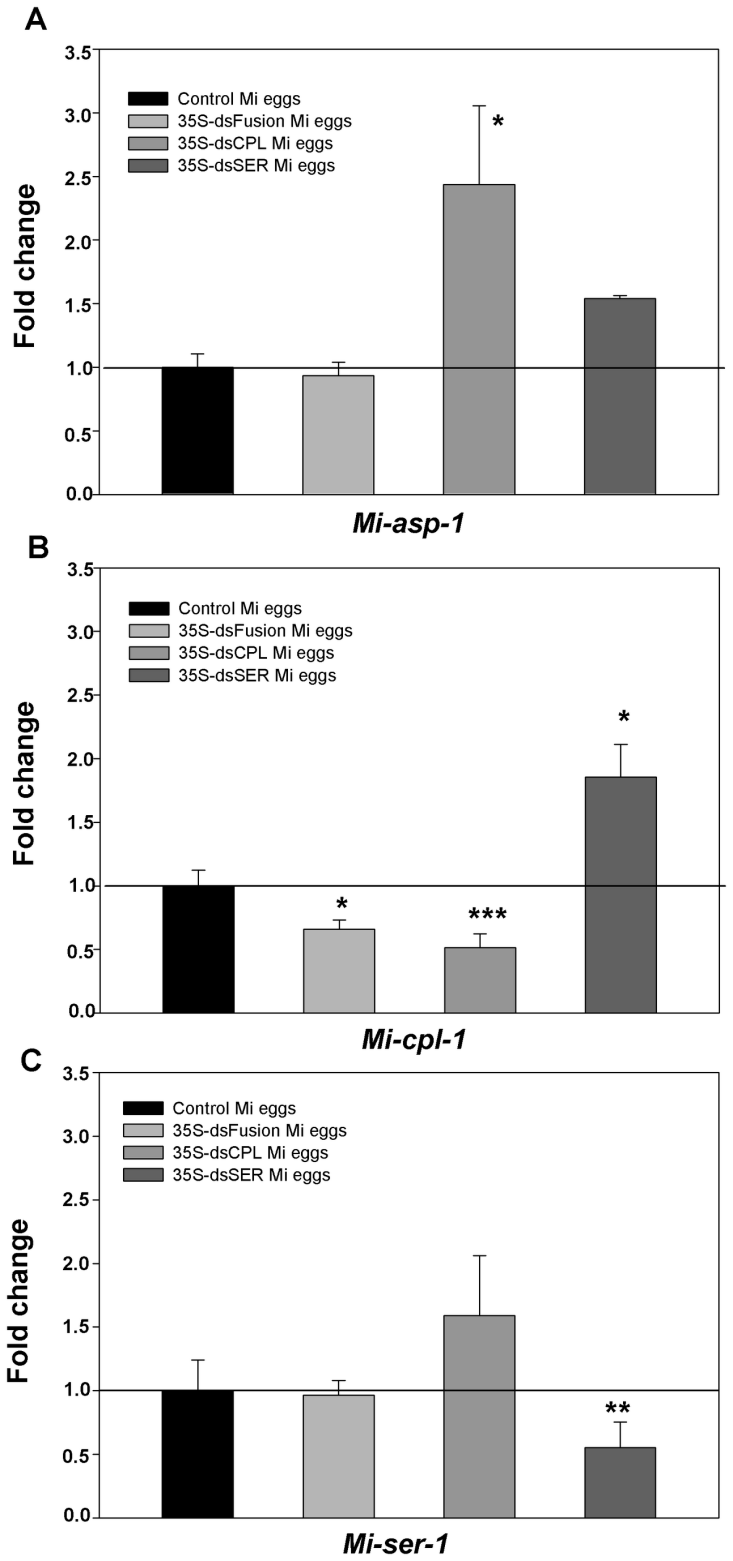
Quantitative RT-PCR showing the transcript levels of proteases in eggs exposed to dsRNAs. Eggs were collected from *M. incognita* females that infected transgenic tobacco lines expressing dsRNA for Mi-SER, Mi-CPL and Mi-ASP, Mi-SER and Mi-CPL fused (fusion). (A) Analysis of *Mi-asp-1*; in *M. incognita* (Mi) eggs of 35S-dsCPL and 35S-dsSER plants *Mi-asp-1* was not exposed to a specific dsRNA. (B) Analysis of *Mi-cpl-1* gene; in Mi eggs of 35S-dsSER plants *Mi-cpl-1* was not exposed to a specific dsRNA. (C) Analysis of *Mi-ser-1* gene; in Mi eggs from 35S-dsCPL plants *Mi-ser-1* was not exposed to a specific dsRNA. Significant differences were assessed by Iteration test (REST Software) where proteases gene expression in nematodes eggs from different plants lines were compared to control plants (*, **, *** = P ≤ 0.05, 0.01 and 0.001, respectively).

To analyze the possible change in transcript levels in nematodes caused by dsRNA and/or siRNA ingestion, total RNA was extracted from eggs originated from females fed on dsRNA or control plants (Figure 6). This RNA was used to perform qRT-PCR and data were obtained from two experiments with three replicates each.

No significant variation on *Mi-asp-1* transcripts levels was observed in eggs from nematodes that have fed on plants expressing dsRNA fusion, for the three proteases studied (dsFusion plants) (Figure 6A). Similar results were seen in eggs that hatched from dsSER plants. Interestingly, transcript levels of *Mi-asp-1* in eggs that came from dsCPL plants increased approximately 2.5 times in comparison with control plants. 

A 50% reduction in *Mi-cpl-1* transcript levels in eggs originating from plants containing the dsFusion and dsCPL was observed (Figure 6B). This reduction in *Mi-cpl-1* transcript levels could be correlated with the reduction in the number of eggs per gram of root (Figure 5A). Interestingly, the transcript level of *Mi-cpl-1* in eggs from dsSER plants increased approximately twice compared to the control.

Finally, no difference was observed on transcript levels of *Mi-ser-1* in eggs that hatched from dsCPL plants (Figure 6C). Similar results were observed in eggs from females fed on dsFusion plants. A 50% reduction of *Mi-ser-1* transcript levels in dsSER eggs was detected. This decrease could explain the reduction in number and viability of eggs from females fed on dsSER plants (Figure 5A and 5B).

### *M. incognita* progeny virulence is affected by protease knock-down

To examine the possibility of additional effects besides those described for parental generation, we decided to evaluate the virulence of J2 originated from females that have fed in *M. incognita* protease dsRNA plants. Therefore, approximately 800 hatched-J2 from females of the three constructs (dsFusion, dsCPL and dsSER) and control ppJ2 were inoculated onto wild-type tobacco (control plants). To evaluate the ability to infect and develop, number of galls and of egg masses was counted. 

**Figure 7:**
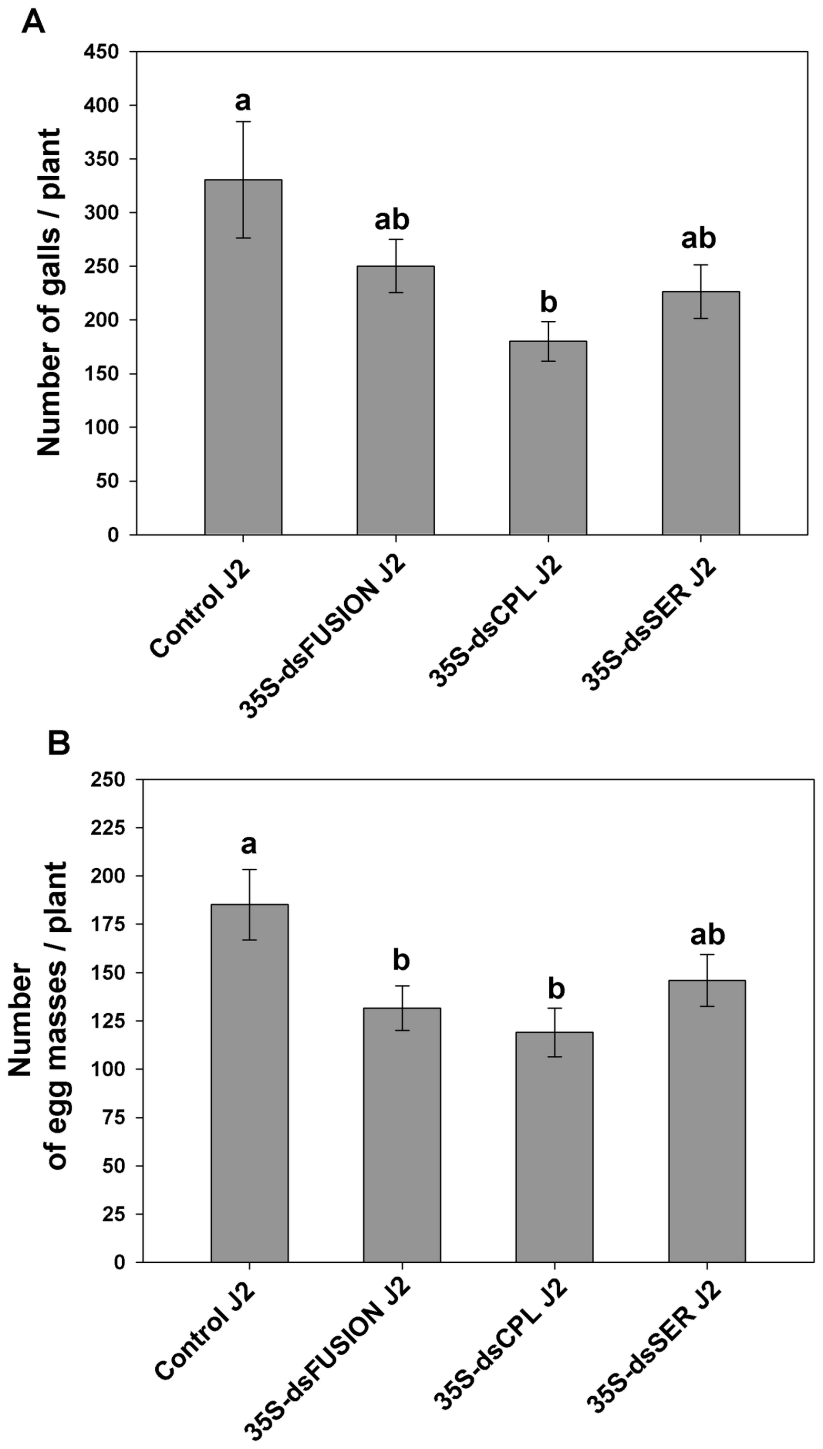
Effect of protease knock-down on progeny virulence of *M. incognita*. Hatched J2 from eggs that were laid by females that feed on transgenic and control plants were inoculated in wild-type tobacco. (A) Relative number of galls per plant at 45 DAI; (B) Relative number of egg masses per plant at 45 DAI; Experiments were repeated twice. Different letters mean statistical significance through one-way ANOVA and Tukey test (P≤0.05).

Although, data on number of galls per plant of J2 from dsFusion did not show statistically difference when compared to control J2 (Figure 7A) (ANOVA, F_3,15_=3.261, *p*=0.05), number of egg masses generated by J2 was 30% less (*p*<0.05) compared to control (Figure 7B) (ANOVA, F_3,32_=3.665, *p*=0.022). Subsequently, number of galls induced by J2 originating from dsCPL plants was evaluated and showed reduction in gall number of 45% (*p*<0.05). Similarly, number of egg masses layed by dsCPL J2 was 32% less (*p*<0.05) compared with the number generated by Control J2. Apparently, this reduction of virulence of J2 from dsFusion and dsCPL correlates with the decreased transcript levels of *Mi-cpl-1* in eggs that originated from these plants.

Finally, when we evaluated the virulence of J2 originating from dsSER females, number of galls and egg masses were not statistically different from control plants. Therefore nematode virulence seemed not to be affected.

## Discussion

The publication of the *M. incognita* genome [9] provided a huge source of data allowing the better understanding of all factors related to nematode parasitism. For example, it was found that this nematode possesses a variety of all representative classes of proteases described in the literature [8]. In addition to already known functions like ordinary digestion of proteins to ingest food, it has been shown that these proteases are involved in several common metabolic pathways as well as in specialized physiological processes like moulting [43] and embryogenesis [44,45]. More recently their potential implication as parasitism effectors, possibly degrading host defense proteins in the plant apoplast, was illustrated by Vieira et al. [14]. A similar role for proteases from the plant pathogenic bacterium *Pseudomonas syringae* was found in which the release of bacterial proteases in plant cells would trigger suppression of plant defense pathways [46]. 

Based on the diversity of the biological functions performed by proteases identified in pathogens, we studied three proteases of *M. incognita*: an aspartic protease, a serine protease, and a cysteine ​protease, to determine their possible involvement during initial steps of nematode infection, feeding site maintenance and nematode development till the reproductive stage. Therefore for each protease, the expression profile, gall morphology, nematode development, reproduction ability, as well as progeny virulence in plants expressing the corresponding knock-down dsRNAs was performed.

### Protease transcript levels vary during nematode development

Our comparative analysis of aspartic, serine and cysteine proteases in the EST databank showed a higher number of cysteine proteases in comparison with the two other classes. These results corroborate with the number of genes of aspartic, cysteine and serine proteases in the *M. incognita* genome [8]. We also detected ESTs of the three proteases in all nematode developmental stages with exception of *Mi-ser-1*, not detected throughout juvenile stages. These data is in agreement with data here shown of qPCR where *Mi-ser-1* transcript level in ppJ2 was almost 7 times less than in eggs. However, we found the highest expression in parasitic juveniles (pJ2/J3/J4), suggesting that this enzyme might also be active in stages where nematodes feed and develop.

Transcript accumulation of proteases by quantitative PCR (qRT-PCR) demonstrated that the three proteases studied show differential accumulation patterns throughout nematode development. Aspartic protease (*Mi-asp-1*) showed highest transcript accumulation levels in parasitic juveniles. This result differed from obtained by Fragoso et al. [13] possibly due to the higher sensitivity of qRT-PCR technique. Previous RT-PCR data demonstrated highest accumulation levels in females [13]. In addition, all data obtained in this study were submitted to statistical analysis, and no differences were observed between expression levels of eggs, ppJ2 and females. Besides, *Mi-asp-1* transcripts are more present in parasitic juveniles and former analysis predicted the presence of a signal peptide in this protease [13]. This suggests that *Mi-asp-1* is likely to be exported out of the cell. These results imply that this aspartic protease might be involved not only in ordinary digestion for nutrition, but also in other unknown aspects during plant-nematode interaction. 

Similarly, serine protease transcripts were present in higher concentrations in juvenile parasitic stages, at moderate levels in eggs and females, and low levels were detected in pre-parasitic juveniles. Although this data corroborate with reported by Fragoso et al. [12], they observed that protease transcripts are more abundant in maturing females containing eggs. A possible role for *Mi-ser-1* beyond ordinary digestion could therefore be attributed to processes related to egg development and embryogenesis

Expression profiling of the nematode cysteine protease *Mi-cpl-1* revealed that transcript levels in eggs and parasitic juveniles (pJ2/J3/J4) were identical (p>0.05), but different from ppJ2 and females. These results differed from data reported by Neveu et al. [10] who observed by RT-PCR that *Mi-cpl-1* had higher expression in ppJ2 and females compared to eggs. Moreover, Shingles et al. [11] observed no difference in transcript levels between ppJ2 and females. These discrepancies might result from a lack of precision when using RT-PCR procedures. We believe that data generated by qRT-PCR allows more accurate transcript quantification. Previous mRNA *in situ* hybridization results have shown that this enzyme is expressed in ppJ2 [10] and females [11] within the intestine. Taken together, these data indicates that this cysteine protease has a more ubiquitous function or exhibits different roles during different stages of nematode development.

### Feeding on plants expressing *M. incognita* dsRNA proteases affects nematode female proportion, reproduction, egg viability and progeny virulence

The success of phytoparasitic nematodes like *M. incognita* largely depends on the efficient use of a vast arsenal of parasitism molecules to circumvent plant defenses while keeping feeding sites functional. Therefore, we investigated here whether the *in planta* reduction of transcripts of three protease genes, individually or simultaneously, would diminish the success rate of nematodes in their host. Even if a similar approach has already been exploited *in vitro* [11] with *Mi-cpl-1*, we present for the first time data silencing nematode proteases, *in planta* affecting nematode propagation. 

We observed that dsRNA lines for Mi-Ser-1, Mi-Cpl-1 and three proteases together (dsFusion) caused a significant reduction in the number of eggs per gram of roots. Gene silencing of a sperm protein (msp) has been reported by Steeves et al. [47] to lower fertility of male *H. glycines* leading to egg reduction (per gram of roots) of up to 68%. Silencing of ribosomal protein genes also resulted in decreased nematode fertility in *H. glycines*, where reduction of egg number per gram of root was up to 87% [30]. Shingles et al. [11] reported the effect of *Mi-cpl-1* gene silencing by *in vitro* soaking resulting in reduction of female size possibly due to nutritional deficiency caused by Mi-Cpl-1 enzyme depletion. Thus, nutritional deficiency could also explain the reduced number of eggs showed here.

We have observed a delay in egg hatching for dsSER plants, indicating that knocking down this gene could interfere in the process of egg development. Fanelli et al. [48] have observed a similar phenotype when partially silencing the chitin synthase gene by soaking *Meloidogyne artiellia* eggs *in vitro* using dsRNA. Matsunaga et al. [49] also knocked down the *Mi-pos-1* gene by soaking, resulting in reduced egg viability in *M. incognita*. Knocking-down Ce-CPL-1 [45] in *C. elegans* by soaking caused embryogenesis defects leading to non-viable eggs. Our results suggest that the Mi-Ser-1 protease could be involved as well in *M. incognita* embryogenesis.

 We further evaluated if the effects of feeding on dsRNA plants can still be continued in the next generation when infecting wild-type plants. Indeed, nematodes in which fed on dsRNA plants did not infect well wild-type plants, produced less galls and egg masses when using dsCPL-J2s. Reduced egg masses were also obtained when dsFusion-J2s were used. J2 from dsSER plants, which hatched normally, were able to infect wild-type plants. 

Consistent data on the effects of RNAi on Root-Knot or Cyst Nematodes offspring are currently lacking. One report by Dubreuil et al. [36] illustrated that *M. incognita* J2 (progeny) infecting *Nicotiana benthamiana* plants infiltrated with TRV virus for gene silencing (VIGS) of calreticulin (TRV::CRT), produced fewer galls in wild-type tomatoes. As such, results presented here describe for the first time the effect of RNAi delivery by plants in RKN progeny revealing the virulence of nematode offspring.

### Protease knock-down can affect target and non-target protease genes in nematode progeny

 In order to get more insight into the long term effects of gene knock-down on nematode development and virulence, eggs laid by nematodes that infected plants expressing proteases dsRNA were collected and analyzed until the end of their life cycle as well as for next generation offspring. We could show a substantial reduction in protease transcript levels in eggs from nematodes that fed on plants expressing homologue dsRNA. Examples are eggs of dsCPL plants females (less *Mi-cpl-1* transcript) and eggs from dsSER plants females (less *Mi-ser-1* transcript). Similar long-term effects of RNAi were evidenced in cysts of *H. glycines* [30] where genes involved in nematode replication and fitness were silenced till five weeks after inoculation. Here, eggs were harvested six weeks after inoculation. Surprisingly, in eggs harvested from dsCPL and dsSER infected plants, non-target proteases genes were up-regulated when the target gene was knocked-down, like *Mi-asp-1* in eggs from dsCPL and *Mi-cpl-1* in eggs from dsSER. This phenomenon of RNAi off-target effect [50] was never described for *M*. incognita although reported before for human cultured cells illustrating the increased expression of certain non-target genes [50]. For insects, the reduced expression of inhibited proteases appears to activate other enzymes belonging to the same or different catalytic class [51-53]. A similar mechanism might explain the effects observed here for nematodes suggesting the existence of a natural and more general response of organisms to compensate for the lack of some proteases. 

 To improve the effectiveness of targeted gene knock-down, we tested a novel strategy to silence different catalytic class of proteases using a single dsRNA expression cassette where we expected the simultaneous knock-down of transcripts of the three proteases targeted. Surprisingly only *Mi-cpl-1* was down-regulated. Moreover, *Mi-ser-1* transcript levels did not change and the unexpected up-regulation of *Mi-asp-1* did not occur like when only *Mi-cpl-1* was knocked-down. Therefore, knocking-down *Mi-cpl-1* and *Mi-ser-1* at the same time from a single dsRNA was clearly less effective than using dsRNA for each protease separately.

 Bakhetia et al. [54] evaluated the simultaneous silencing of dg13 and dg14 genes of *H. glycines* using the soaking technique and found an unexpected increase in dg13 transcript abundance; a phenomenon hereafter called RNA amplification (RNAa). This RNAa effect was only detected so far in mammalian cells when using a promoter as dsRNA target [55,56]. It is not clear whether silencing proteases could be less effective in dsFusion plants due to the larger size of dsRNA (~600 bp) as compared to (~200 bp) dsCPL and dsSER constructs, or due to a simple dose effect. A previous report show that fewer dsRNA/siRNA results in less effective silencing [57]. Crossing dsCPL and dsSER lines could therefore be considered as an alternative strategy to circumvent the difficulties encountered. Though, for proteases, simultaneous silencing of the same dsRNA construct might not be a promising strategy to effectively silence multiple genes. Moreover, the off-target effects, like the up-regulation of non-target proteases give no significant effects on nematode development. 

It seems that durable strategies to control nematode virulence will probably rely on the silencing of different well chosen nematode target genes preferentially belonging to different gene classes and coordinately expressed in a single host plant.

## Conclusions

Here we report that overexpressing dsRNA *in planta* for different *M. incognita* proteases exerts long-term effects in nematode progeny such as egg number reduction. Depending on the protease down-regulated, we noticed difficulties with nematode progeny hatching by knocking down *Mi-ser-1* and disturbance to infect a new host plant when knocking down *Mi-cpl-1*. Although animal proteases are known to be important for protein digestion mainly for feeding and nutrition, they can play different roles in a many biological processes. Based on the data presented here we can conclude that at least two of the three proteases studied are possibly implicated in nematode reproduction, embryogenesis and offspring virulence. They are therefore attractive targets to engineer plants able to deliver nematode proteases dsRNA during infection. The cell specific knock-down of such proteases using promoters with strong preference for feeding cells, and also pyramiding with other nematode essential genes, could therefore be an attractive strategy to generate new tools for phytonematode control. 

## Supporting Information

Figure S1 **Transgenic plants expressing dsRNA for *M. incognita* proteases do not affect morphology and size of nematode feeding site.** (A) Bright-field images from giant cells after toluidine blue-staining of *N. tabacum* control and RNAi lines galls, 14 DAI with *M. incognita*. n: nematode; * : giant cell. bar, 100 µm. (B) Giant cell surface (µm^2^) of wild-type plants and of 35S-dsFUSION, 35S-dsCPL and 35S-dsSER overexpressing lines were measured at 14 DAI. Measurements were made on a minimum of 21 giant cell sections (only the 2 - 3 largest giant cells were measured per gall). There was no difference between the treatments by one-way ANOVA test (F_3,100_=1.033; *p*=0.381).
(TIF)

Figure S2 **Number of galls and egg masses in nematode infected proteases RNAi lines.** (A) Relative number of galls per plant. (B) Statistical analysis was carried out by one-way ANOVA and Tukey test (*p*≤0.05).
(TIF)

Table S1 **Number of ESTs of proteases from *Meloidogyne incognita* at different developmental stages.**
(XLSX)
