# Supplementary figures and images for: Knocking-Down Meloidogyne incognita Proteases by Plant-Delivered dsRNA Has Negative Pleiotropic Effect on Nematode Vigor
Source: PLoS One. 2013 Dec 31;8(12):e85364. doi: 10.1371/journal.pone.0085364 (PMC3877404; doi:10.1371/journal.pone.0085364)

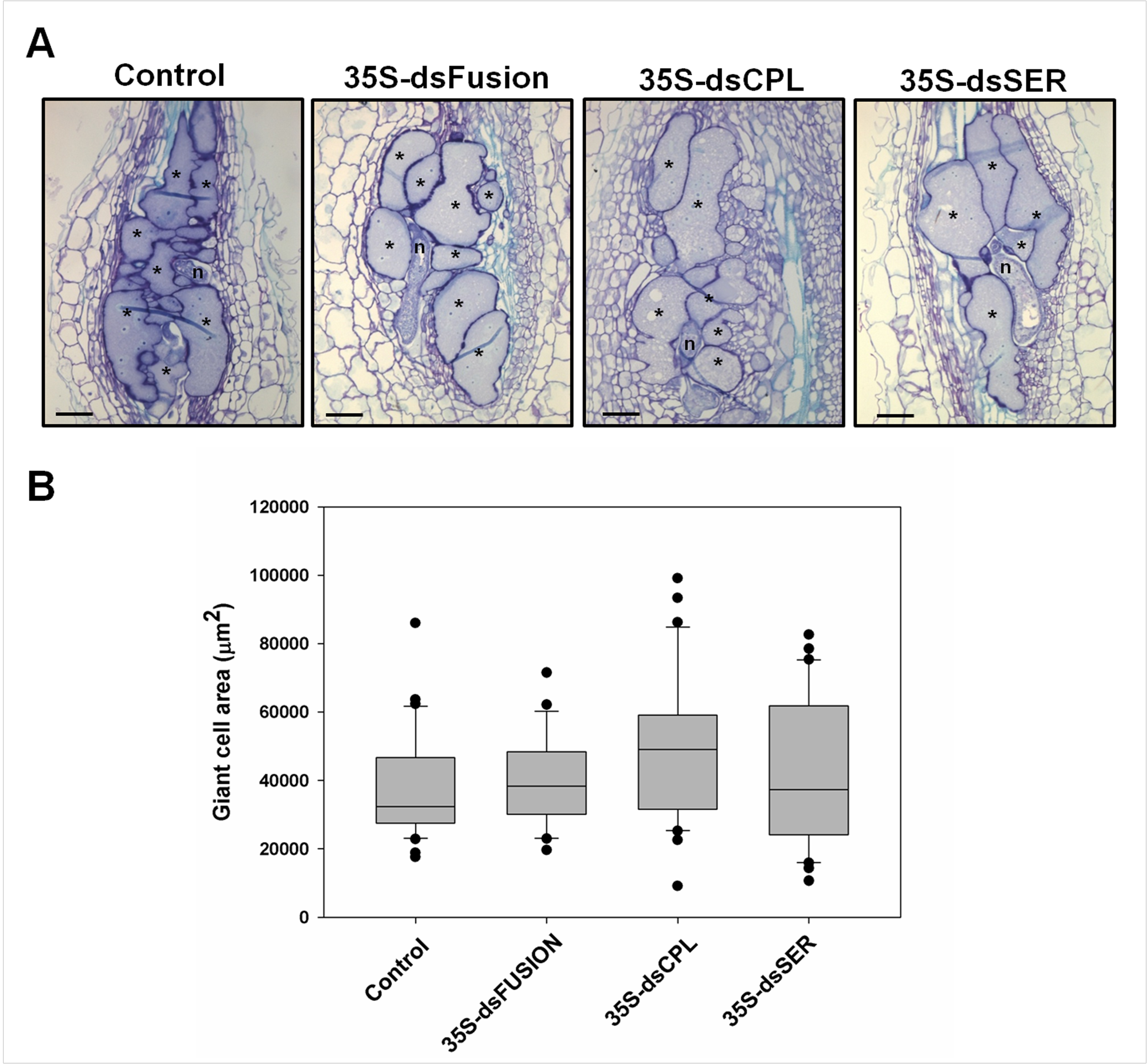

Supplement: Figure S1 — Transgenic plants expressing dsRNA for M. incognita proteases do not affect morphology and size of nematode feeding site. (A) Bright-field images from giant cells after toluidine blue-staining of N. tabacum control and RNAi lines galls, 14 DAI with M. incognita. n: nematode; * : giant cell. bar, 100 µm. (B) Giant cell surface (µm2) of wild-type plants and of 35S-dsFUSION, 35S-dsCPL and 35S-dsSER overexpressing lines were measured at 14 DAI. Measurements were made on a minimum of 21 giant cell sections (only the 2 - 3 largest giant cells were measured per gall). There was no difference between the treatments by one-way ANOVA test (F3,100=1.033; p=0.381). (TIF) [file pone.0085364.s001.tif]

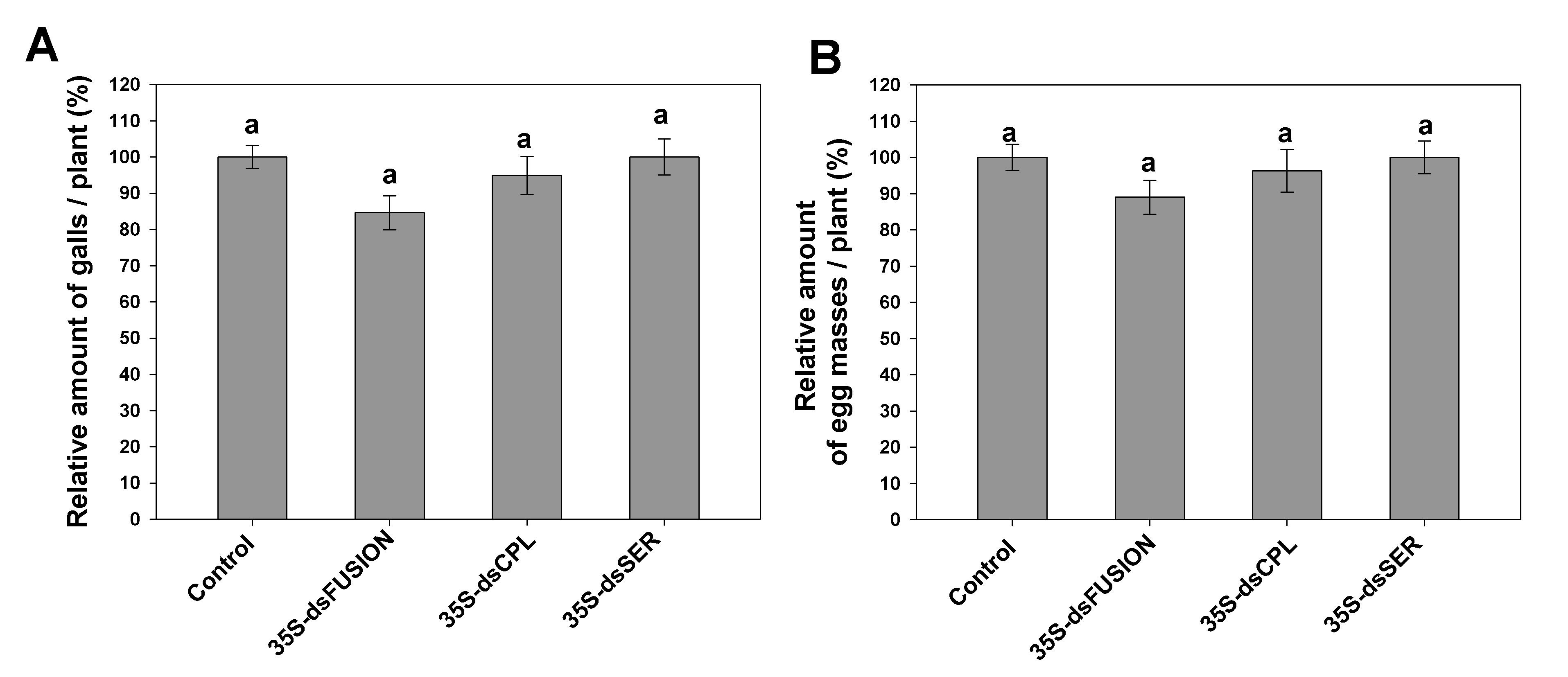

Supplement: Figure S2 — Number of galls and egg masses in nematode infected proteases RNAi lines. (A) Relative number of galls per plant. (B) Statistical analysis was carried out by one-way ANOVA and Tukey test (p≤0.05). (TIF) [file pone.0085364.s002.tif]
